# Application of piecewise VMAT technique to whole-brain radiotherapy with simultaneous integrated boost for multiple metastases

**DOI:** 10.1186/s13014-022-02059-6

**Published:** 2022-05-07

**Authors:** Yuan Xu, Yingjie Xu, Kuo Men, Jianping Xiao, Jianrong Dai

**Affiliations:** Department of Radiation Oncology, National Cancer Center/National Clinical Research Center for Cancer/Cancer Hospital, Chinese Academy of Medical Sciences and Peking Union Medical College, Beijing, 100021 China

**Keywords:** Piecewise VMAT, WBRT, SIB, Brain metastases, HT

## Abstract

**Purpose:**

This study implemented a piecewise volumetric modulated arc therapy (P-VMAT) for realizing whole-brain radiation therapy (WBRT) with simultaneous integrated boost (SIB) for multiple brain metastases (> 40 metastases) with a conventional C-arm linear accelerator.

**Materials and methods:**

This study retrospectively analyzed 10 patients with multiple brain metastases (40–120 metastases, median 76), who underwent WBRT and SIB using helical tomotherapy (HT). The prescribed doses were 40 Gy/20 f and 60 Gy/20 f for WBRT and SIB, respectively. Corresponding new HT plans were designed with P-VMAT using 7 arcs. For each arc, the collimator was rotated to 45°, and the field width was limited to 2.5 cm with 0.5 cm overlap with adjacent arcs. Thus, each arc covered only one section of the brain target volume. A conventional dual arc VMAT (DA-VMAT) plan was also designed. HT, P-VMAT, and DA-VMAT plans were compared using dose distribution reviews and dosimetric parameters. ArcCHECK phantom measurements were performed for verification of P-VMAT plans.

**Results:**

No significant differences in the mean coverage of the whole-brain target and metastases were observed between HT and P-VMAT (*p* > 0.05). The conformity index for the whole-brain target improved with P-VMAT compared with HT (*p* < 0.05). Furthermore, the volume of 44 Gy V_44_ (110% of prescribed dose for WBRT) received for whole-brain significantly reduced with P-VMAT from 38.2 ± 12.9% to 23.3 ± 9.4% (*p* < 0.05), and the maximum dose for organs at risks such as the hippocampus, optical nerve, optical chiasm, and spinal cord declined with P-VMAT (*p* < 0.05). Unlike HT and P-VMAT, DA-VMAT was clinically unacceptable because V_44_ in the whole-brain was too high (54.7 ± 8.2%). The mean absolute dose gamma passing rate for P-VMAT plans was 97.6 ± 1.1% (3%/3 mm criterion, 10%).

**Conclusions:**

P-VMAT is favorable for WBRT and SIB for multiple brain metastases. It provides comparable coverage of whole-brain target and SIB, with better conformity, lower V_44,_ and better dose sparing of organs at risk compared with HT. Furthermore, results show that DA-VMAT fails clinical practice even for a relatively large number of brain metastases with a high degree of plan complexity. The patient specific verification demonstrates the feasibility of P-VMAT for clinical application.

## Introduction

Brain metastases are common intracranial tumors in approximately 20–40% of patients with cancer [1] while multiple brain metastases accounted for about 70% [2]. Whole-brain radiation therapy (WBRT) is a standard modality for treating brain metastases [3]. However, the median survival is about 3–6 months for WBRT [4]. Stereotactic radiosurgery (SRS) or stereotactic fractionated radiotherapy (SRT) is a favorable approach for treating few metastases. WBRT is concerned with learning and memory function decline and no benefit of survival [5, 6]. Nevertheless, SRS alone has a high rate of remote disease progression, and developing new lesions increases with the increased number of brain metastases [7]. With the development of intensity-modulated radiotherapy (IMRT), WBRT with simultaneous integrated boost (SIB) or sequential SRS/SRT were introduced and proved to be effective for treating brain metastases [8, 9]. Comparing with WBRT followed by sequential SRS/SRT, WBRT with SIB has better dose distribution to spare normal tissue and organs at risk (OAR) and a reduced treatment period [9]. It was reported that for 43 patients with metastases ranging from 3 to 36, the median survival time was 21.3 months treated by WBRT + SIB using helicon tomotherapy (HT) which was obviously longer than WBRT alone [10]. Therefore, for radiation therapy of many brain metastases, WBRT plus SIB is also recommended to increase local control [10].

WBRT with SIB has employed several techniques, such as IMRT, volumetric modulated arc radiotherapy (VMAT), and HT are common choices [11, 12]. However, conventional IMRT or dual arc VMAT (DA-VMAT) delivered with a C-arm linear accelerator treated a limited number of brain metastases. Brain metastases vary between tens or over 100 for some patients. The complexity of plan optimization increases with increased metastases and is difficult to solve using the inverse optimizer in the treatment planning system for IMRT or VMAT. For intracranial SRS/SRT, non-coplanar beams/arcs are commonly used as non-coplanar beams/arcs increase the conformity and dose fall-off outside the target area [13]. For WBRT, it was also reported by a few publications that WBRT had the benefit of reducing the dose delivering to some OARs such as parotid or hippocampus [14, 15]. Nevertheless, non-coplanar technique is still not a common treatment modality for WBRT as it will largely increase the treatment time for WBRT with 10–20 fractions. HT is clinically used for treating large-scale brain metastases like with WBRT + SIB in our institution. However, HT is not available in many radiotherapy centers. Meanwhile, when treating large-scale brain metastases, sparing normal brain tissue should be improved to protect the neurocognition in patients [16].

Most studies reported the use of WBRT + SIB with 1–3 brain metastases [17, 18]. The radiotherapy technology of WBRT + SIB with a large number of brain metastases (> 40) is not reported. Exploring the feasibility of treating a large number of brain metastases with conventional C-arm linac, a new technique called piecewise volumetric modulated arc therapy (P-VMAT) was applied in this study. Recently, the technique was proposed by our group for WBRT with hippocampal sparing to improve plan quality and better spare hippocampus [19, 20]. This study designed P-VMAT plans for WBRT + SIB with a large number of brain metastases and compared it with HT. DA-VMAT plan was also optimized for each patient for comparison.

## Materials and methods

### Patient information

**Fig. 1.**
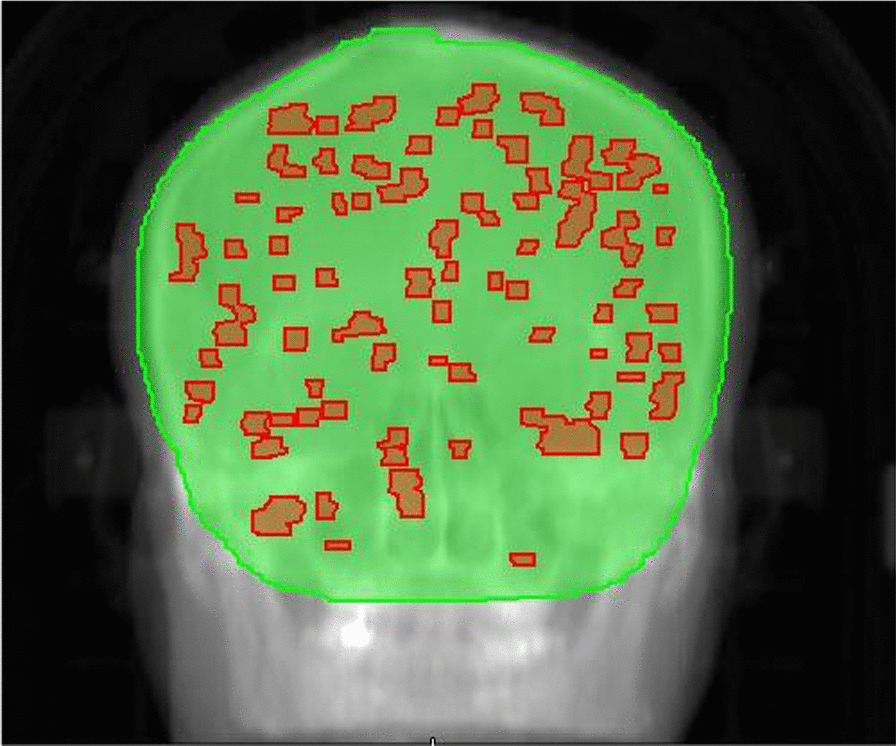
Illustration of a target volume treated with WBRT + SIB for multiple brain metastases (Green: PTV-brain, red: GTV)

This retrospective study was approved by the review ethical board of our institute, and informed consent was waived. Ten patients with multiple brain metastases treated using HT were included in the study. The mean patient age was 48.7 (range 27–71) years. Table 1 shows the number of brain metastases varying between 40 and 120 (median 76). Patients were scanned by contrast-enhanced computed tomography (CT) with a brilliant CT big bore (Philips Healthcare, Best, Netherlands) using 2 mm thick slices, and fused with contrast-enhanced magnetic resonance imaging (MRI) scanner (Philips Healthcare, Best, Netherlands) with the same thickness. Gross target volume (GTV) was defined as the enhanced metastatic region in MRI T_1_ enhanced sequence, excluding edema area, numbered GTV1, GTV2 etc. for each metastasis. And GTV is the combination of all metastases. The clinical target volume (CTV) of the brain (CTV-brain) comprised the whole-brain. The planning target volume PTV-brain was generated by expanding a 5 mm margin to CTV-brain, and hippocampus with a 3 mm margin expanding in three dimensions was excluded from PTV-brain. A typical target volume is shown in Fig. 1. The total volume of all GTV for each patient varied from 3.7 to 45.1 cc (mean 17.4 ± 13.3 cc), and the volume for PTV-brain ranged from 1443.1 to 2068.8 cc (mean 1762.1 ± 198.1 cc). The OARs were contoured on the CT images, which included lens, hippocampus, optical nerve, optical chiasm, spinal cord, brain stem and pituitary.

**Table 1:** Metastases and PTV-brain description

Patient number	Metastases (*n*)	GTV (cc)	PTV-brain (cc)
1	47	4.54	1443.1
2	107	12.25	1812.4
3	52	14.19	1614.1
4	67	16.95	1745.5
5	84	12.02	1814.7
6	50	3.66	2068.8
7	120	45.05	1859.8
8	40	6.95	1788.7
9	106	25.98	1980.3
10	97	32.59	1493.8

### Treatment planning

#### HT

HT was used for WBRT with SIB for treatment. Patient plans were designed with a Hi-Art planning station (version 5.1) for tomotherapy (Tomotherapy Inc, Madison, Wisconsin). An iterative inverse treatment planning algorithm was utilized for HT planning. The dynamic jaw was used with a field width of 2.51 cm, and a modulation factor of 2.6. The value of pitch was 0.287 with a fine calculation grid of 0.264 cm × 0.264 cm.

#### P-VMAT

**Fig. 2:**
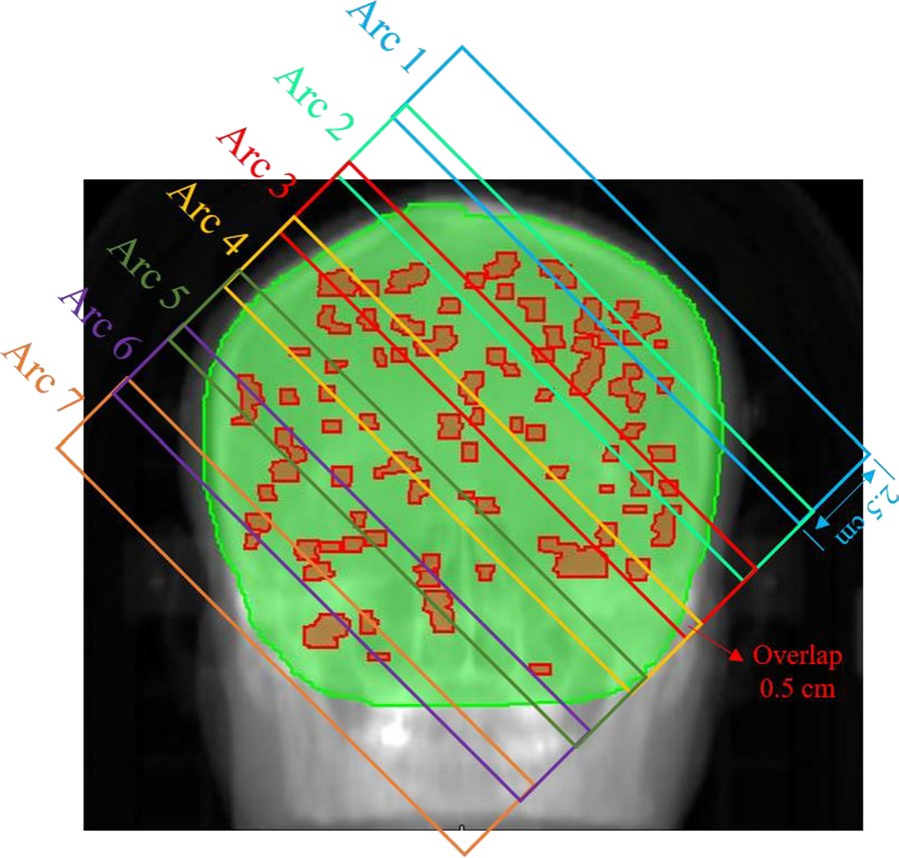
Illustration of the layout of 7 arcs for P-VMAT

The Pinnacle treatment planning system (version 9.1, Philips Healthcare, Eindhoven, Netherlands) redesigned all plans using a 6 MV X-ray delivered using an Elekta Versa HD accelerator (Elekta Oncology Systems, Crawley, UK). The optimization algorithm for VMAT planning was SmartArc. The multi-leaf collimator (MLC) module was Agility with 80 pairs of leaves and leaf width was 5 mm at the isocenter. The dose calculation grid was 0.4 cm × 0.4 cm × 0.4 cm. P-VMAT had seven full arcs with a collimator rotating to 45° and a couch angle of 0° using dynamic jaws. As shown in Fig. 2, the maximum field width was limited to 2.5 cm for each arc, similar to HT. The target volume was covered by seven piecewise arcs from the top to the bottom with 0.5 cm overlap regions between every two adjacent arcs to ensure uniform dose coverage in the overlap regions.

#### DA-VMAT

DA-VMAT plans use two full arcs with opposite rotational directions (clockwise and counterclockwise) without limiting the field width for comparison. The collimator and couch angle were the same to P-VMAT.

#### Plan optimization

The optimization parameters were similar among HT, P-VMAT, and DA-VMAT. It covered 95% of every metastasis with a prescribed dose of 60 Gy delivering in 20 fractions and covered 95% of the PTV-brain with 40 Gy/20 f. A metastasis close to critical OARs, such as brain stem was de-escalated to 50 Gy for preventing the occurrence of neurologic deficit or brain stem necrosis. For OARs, the maximum dose (D_max_) for the lens was less than 9 Gy. The D_max_ for the hippocampus was less than 40 Gy if it was not adjacent to metastases. If the hippocampus was close to metastases, the dose constrain for the maximum dose of hippocampus could be loosen to ensure the coverage of metastases. The D_max_ for the spinal cord was less than 40 Gy, and that for the brain stem was less than 54 Gy. The D_max_ for optical nerve, optical chiasm, and pituitary was lower than 110% of the prescribed dose. The maximum dose in PTV-brain excluding GTV was set lower than 44 Gy.

### Plan evaluation

The representative dosimetric parameters were evaluated for all plans. The conformity indices (CI) of PTV-brain were calculated using Paddick’s formula [21]: CI = (TVPV)^2^/(TV × PV), where TVPV is the absolute volume in PTV-brain covered by the prescription dose 40 Gy, TV is the absolute volume of PTV-brain and PV is the absolute volume covered by the prescription dose 40 Gy inside the body of patient. CI values range between 0 and 1 and CI close to 1 indicated better conformity. The homogeneity index (HI) was defined as [22]: HI = D_5%_/D_95%_, where D_5%_ and D_95%_ are doses covering 5% and 95% of the target volume, respectively, and a smaller HI indicates a better homogeneity. HI was calculated only for GTV since SIB was in PTV-brain, which nullified HI for evaluating homogeneity in the PTV-brain. Hence, V_44_ (the proportion of volume receiving 110% prescribed dose in PTV-brain) indicated homogeneity in PTV-brain. The coverage of GTV V_60_ (the proportion of volume receiving 60 Gy in GTV) and the coverage of PTV-brain V_40_ (the proportion of volume receiving 40 Gy in PTV-brain) were evaluated for all plans. Both maximum dose (D_max_) and mean dose (D_mean_) of the lens, hippocampus, optical nerve, optical chiasm, spinal cord, brain stem, and pituitary were recorded from the evaluation tool in treatment planning system. Monitor unit (MU) for each plan was also recorded, and treatment delivery time was counted by a dry run with the accelerator.

All data were statistically analyzed with SPSS (version 19.0, IBM, New York, USA). An independent sample test was used for analyzing parameters with normal distribution; otherwise, a nonparametric Wilcoxon signed-rank test was used for a statistical test. A value of *p* < 0.05 was considered statistically significant.

### Plan verification

A 3D diode array ArcCHECK phantom (Sun Nuclear Corporation, Melbourne, USA) were used for patient specific verification of P-VMAT plans. The data was analyzed with SNC patient software (v8.2, Sun Nuclear Corporation, Melbourne, USA). The criterion for the absolute dose gamma passing rate is 3%/3 mm with threshold of 10%.

## Results

**Fig. 3:**
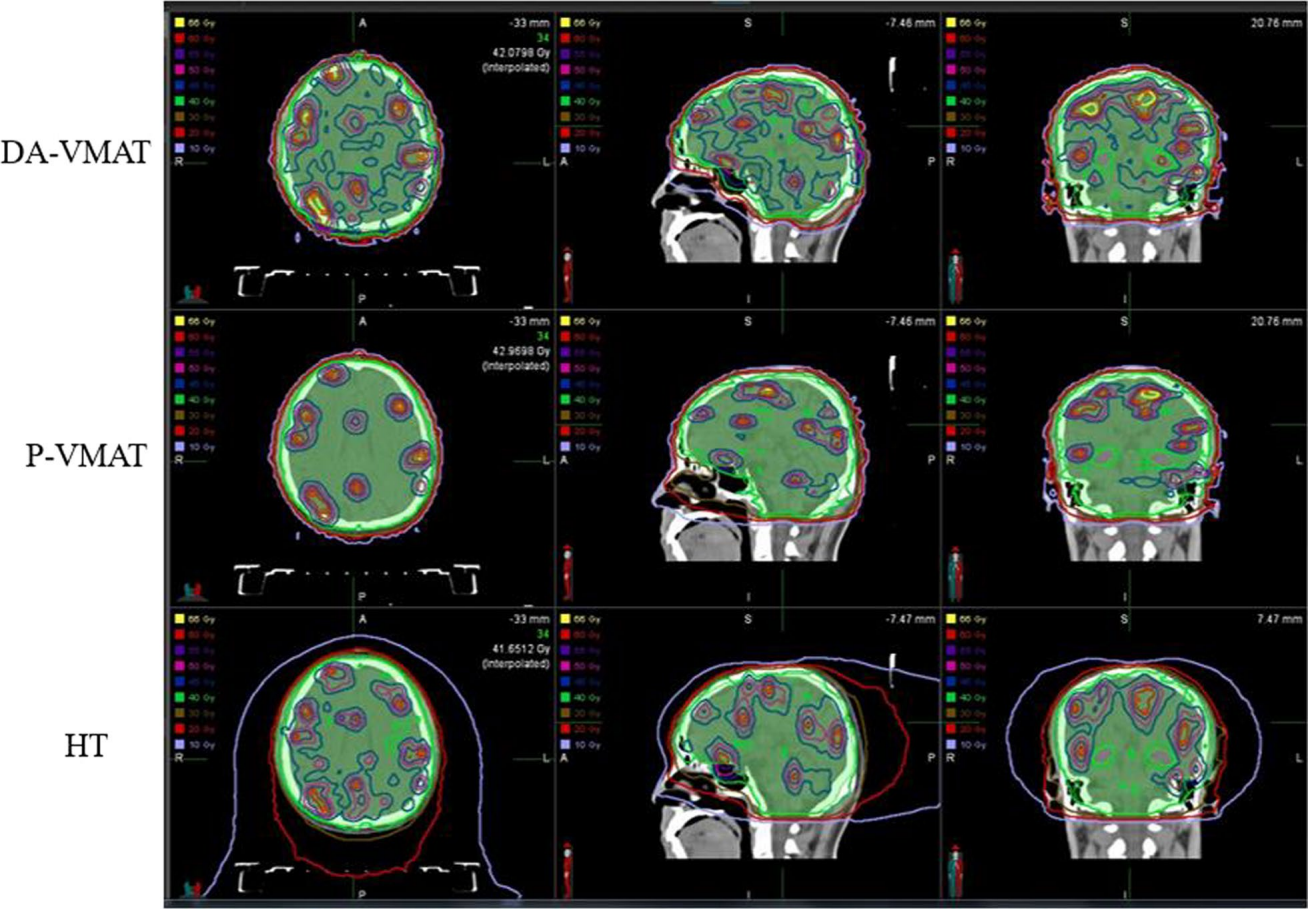
Comparison of dose distribution among HT, P-VMAT and DA-VMAT (colorwash green: PTV-brain, red: GTV)

**Fig. 4:**
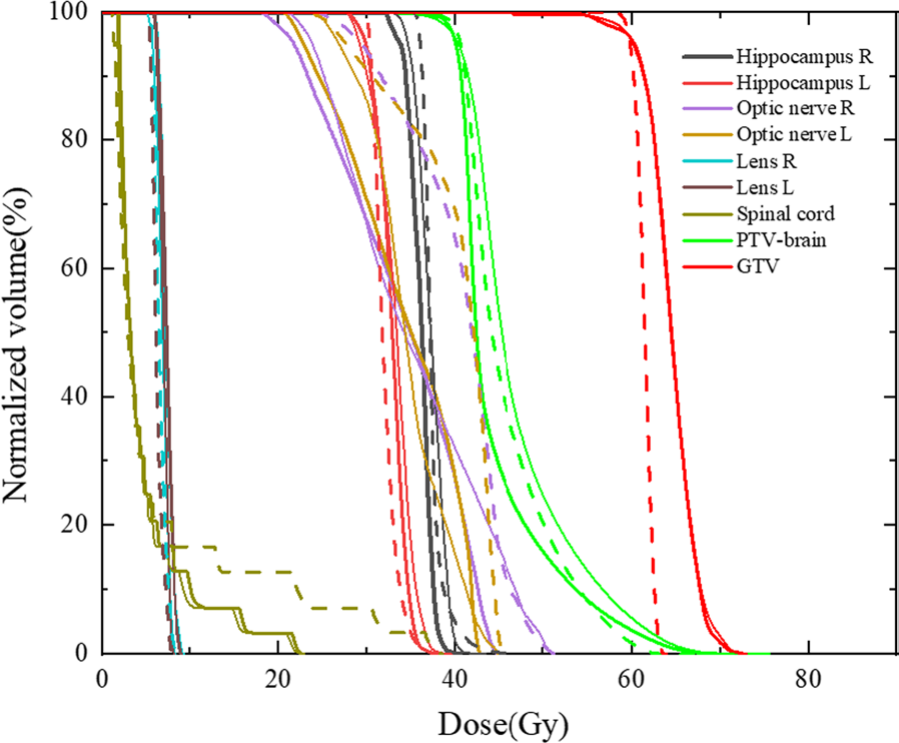
Dose-volume histogram of the target volume and selected OARs. (Dashed line: HT, thick solid line: P-VMAT, thin solid line: DA-VMAT)

Figure 3 shows an example of dose distribution for a patient with 107 brain metastases planning with HT, P-VMAT, and DA-VMAT separately. From the figure, the volume covered by 4500 cGy in PTV-brain was obviously smaller for HT and P-VMAT comparing to DA-VMAT. Thus, the DA-VMAT plan quality was unacceptable for clinical practice because the volume covered by 4500 cGy was too large in normal brain tissue. In Fig. 4, a comparison of the dose-volume histogram also shows that the homogeneity in PTV-brain was best for P-VMAT compared with HT and DA-VMAT.

### Dosimetric evaluation of target volume

All plans were normalized to cover 95% of GTV and PTV-brain with the prescribed dose. The average dosimetric parameters of GTV and PTV-brain planning with three techniques are shown in Table 2 and statistical analyses are illustrated in Table 4. The mean coverage was about 95% for both GTV and PTV-brain, and no significant difference among HT, P-VMAT, and DA-VMAT (*p* > 0.05). Meanwhile, P-VMAT had the highest CI of 0.911 ± 0.031, compared with HT and DA-VMAT. Regarding hot spot V_44_ in PTV-brain, it was measured 23.3 ± 9.4% for P-VMAT and significantly better than HT (38.2 ± 12.9%, *p* < 0.05) and DA-VMAT (54.7 ± 8.2%, *p* < 0.05). The V_44_ in PTV-brain for DA-VMAT was high and shows a poor dose homogeneity in the brain. The mean HI of GTV for HT was 1.092 ± 0.031, better than P-VMAT (*p* < 0.05) and DA-VMAT (*p* < 0.05).

**Table 2:** Comparison of dosimetric parameters (mean ± standard) for target volume among HT, P-VMAT and DA-VMAT

Parameters	DA-VMAT	P-VMAT	HT
PTV-brain			
V_40_(%)	95.5 ± 0.6	95.3 ± 0.4	95.3 ± 0.8
V_44_(%)	54.7 ± 8.2	23.3 ± 9.4	38.2 ± 12.9
CI	0.879 ± 0.018	0.911 ± 0.031	0.832 ± 0.022
GTV			
V_60_ (%)	95.0 ± 0.1	95.1 ± 0.2	95.1 ± 1.8
D_5%_ (Gy)	68.7 ± 2.6	67.1 ± 1.0	65.5 ± 1.6
HI	1.146 ± 0.044	1.119 ± 0.016	1.092 ± 0.031

### OARs

The dosimetric parameters and statistical analyses for OARs are illustrated in Tables 3 and 4, separately. No significant difference exists between HT and P-VMAT for the D_max_ of both the left and right lens (*p* > 0.05). Sparing of the lens was better for DA-VMAT compared with P-VMAT (*p* < 0.05). Compared with HT, both D_max_ and D_mean_ were lower for the hippocampus, optical nerve, optical chiasm, pituitary, and spinal cord with P-VMAT (*p* < 0.05). Moreover, P-VMAT spared most OARs better than DA-VMAT (*p* < 0.05), except for the D_mean_ of the left optical nerve and the D_max_ of the spinal cord (*p* > 0.05) shown in Tables 3 and 4. Furthermore, all dosimetric constraints for HT and P-VMAT were clinically acceptable.

**Table 3:** Comparison of dosimetric parameters (mean ± standard) for OARs among HT, P-VMAT and DA-VMAT

Parameters	DA-VMAT	P-VMAT	HT
Lens L			
D_max_ (Gy)	7.25 ± 0.89	8.26 ± 0.88	8.04 ± 1.34
D_mean_ (Gy)	5.92 ± 0.73	6.73 ± 0.80	6.47 ± 0.74
Lens R			
D_max_ (Gy)	7.29 ± 0.64	8.23 ± 0.53	8.03 ± 0.93
D_mean_ (Gy)	5.90 ± 0.61	6.89 ± 0.33	6.43 ± 0.55
Hippocampus L			
D_max_ (Gy)	44.48 ± 5.34	42.61 ± 5.19	51.49 ± 10.20
D_mean_ (Gy)	36.76 ± 3.34	34.58 ± 2.89	40.66 ± 6.32
Hippocampus R			
D_max_ (Gy)	44.97 ± 4.80	42.23 ± 5.40	50.48 ± 9.16
D_mean_ (Gy)	37.73 ± 3.64	35.43 ± 2.83	40.47 ± 6.12
Optic nerve L			
D_max_ (Gy)	44.56 ± 2.90	41.51 ± 3.58	47.26 ± 4.76
D_mean_ (Gy)	32.68 ± 4.27	32.30 ± 4.48	39.22 ± 2.49
Optic nerve R			
D_max_ (Gy)	45.65 ± 4.12	41.84 ± 3.25	47.18 ± 4.34
D_mean_ (Gy)	33.52 ± 5.21	32.41 ± 5.24	39.66 ± 2.56
Optic chiasm			
D_max_ (Gy)	49.22 ± 3.37	45.47 ± 3.11	51.87 ± 6.54
D_mean_ (Gy)	43.49 ± 1.59	41.08 ± 1.43	43.01 ± 1.18
Spinal cord			
D_max_ (Gy)	30.09 ± 8.51	30.64 ± 8.35	42.35 ± 3.07
Brain stem			
D_max_ (Gy)	53.91 ± 4.75	51.32 ± 5.98	50.74 ± 5.80

**Table 4:** *p* values for statistical analyses of dosimetric parameters among HT, PA-VMAT and DA-VMAT

Parameters	DA-VMAT versus P-VMAT	DA-VMAT versus HT	P-VMAT versus HT
PTV-brain			
V_40_ (%)	0.878	0.878	0.203
V_44_ (%)	0.005	0.007	0.005
CI	0.007	0.007	0.005
GTV			
V_60_ (%)	0.407	0.575	0.721
D_5%_ (Gy)	0.009	0.005	0.007
HI	0.007	0.005	0.013
Lens L			
D_max_ (Gy)	0.005	0.285	0.333
D_mean_ (Gy)	0.005	0.441	0.445
Lens R			
D_max_ (Gy)	0.005	0.445	0.721
D_mean_ (Gy)	0.005	0.093	0.028
Hippocampus L			
D_max_ (Gy)	0.007	0.028	0.013
D_mean_ (Gy)	0.005	0.022	0.009
Hippocampus R			
D_max_ (Gy)	0.005	0.074	0.013
D_mean_ (Gy)	0.005	0.074	0.017
Optic nerve L			
D_max_ (Gy)	0.005	0.022	0.005
D_mean_ (Gy)	0.284	0.005	0.005
Optic nerve R			
D_max_ (Gy)	0.005	0.285	0.005
D_mean_ (Gy)	0.047	0.005	0.005
Optic chiasm			
D_max_ (Gy)	0.005	0.114	0.005
D_mean_ (Gy)	0.005	0.153	0.009
Spinal cord			
D_max_ (Gy)	0.203	0.005	0.005
Brain stem			
D_max_ (Gy)	0.009	0.007	0.959

### MU and treatment times

The average MU values were 5564 ± 364 MU, 4161 ± 379 MU, and 1737 ± 185 MU for HT, P-VMAT, and DA-VMAT, respectively. The average time for treatment delivery was longer (497 ± 34 s) delivering with P-VMAT compared with (230 ± 21 s) for DA-VMAT (*p* < 0.05) and (393 ± 25 s) for HT (*p* < 0.05).

### Plan verification results

The mean absolute dose gamma passing rate for P-VMAT plans was 97.6 ± 1.1% (3%/3 mm criterion, 10%), and the passing rates for all P-VMAT plans were larger than 95% which fulfill the requirements for clinical practice.

## Discussions

P-VMAT is an alternative to HT for treating a large number of brain metastases with comparable or improved plan quality. Conventional VMAT using 7 arcs was also tried without limiting the field width to compare with P-VMAT which limited the field width for each arc. However, the plan quality did not improve compared with DA-VMAT and not fulfill the dose constraints or the treatment planning system had no solution. It means that the improvement of plan quality for P-VMAT was not because the number of arcs increase, but for limiting the field width for each arc. Thus, the reason for the superior P-VMAT can be explained as follows:

A local gradient-based optimization method was adopted in the algorithm named SmartArc for Pinnacle [23, 24]. In principle, the optimization results achieved with P-VMAT is a subset of conventional VMAT using dynamic jaws without limiting the field width. However, due to the constraints of delivery time, machine-specific parameters, limited motion speed of jaw positions and optimization algorithm, it is difficult for the treatment planning system to get a solution similar to P-VMAT without indicating jaw positions. For partial target volume covered by each arc, the modulation ability was improved as the motion of the MLC was limited to a small range. Hence, it is beneficial for P-VMAT by indicating jaw positions for partial regions manually. HT is similar to a CT scanner with a linac replacing the X-tube [25]. The target volume is irradiated using a fan beam with rotating linac continuously moving the treatment couch. With this special helical design, HT can achieve comparable or better plan quality than conventional VMAT, which is used for a limited number of brain metastases [26]. Several studies compared HT and VMAT for different sites, and mixed results were reported [27–29]. However, these studies show that HT has a larger lower dose-volume than VMAT, which showed a relatively slow dose fall-off for HT. For treating a larger number of brain metastases with WBRT + SIB, the dose gradient varied swiftly in the target volume. Therefore, a better plan quality could be achieved with P-VMAT compared with HT, when similar field width (2.5 cm) was used.

**Fig. 5:**
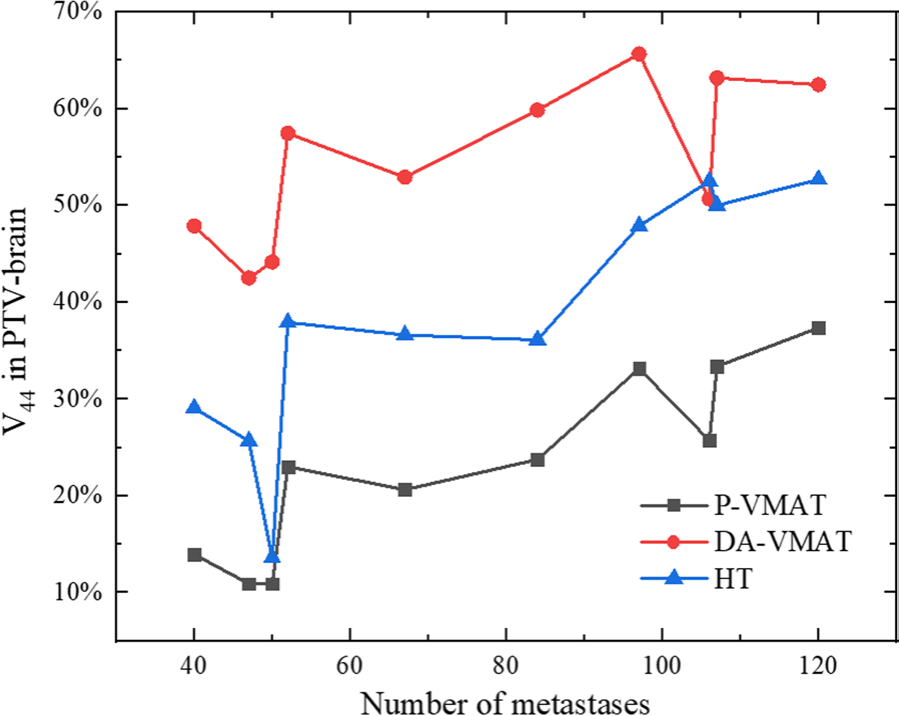
V_44_ in PTV-brain varies with the number of metastases among HT, P-VMAT and DA-VMAT

**Fig. 6:**
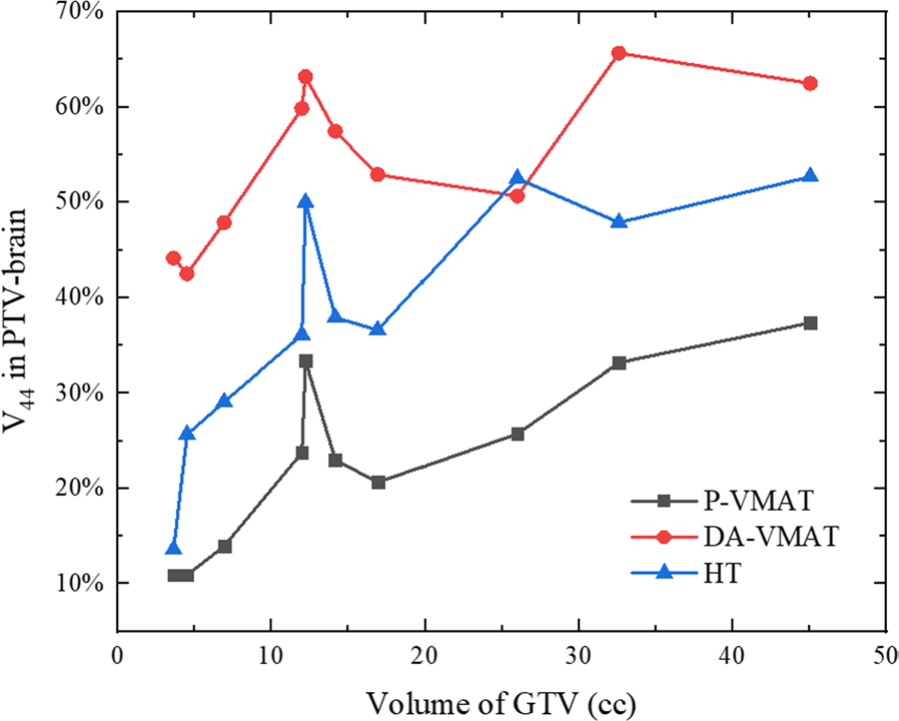
V_44_ in PTV-brain varies with the volume of GTV among HT, P-VMAT and DA-VMAT

Several studies have reported declines in functions of learning and memory associated with irradiation of the hippocampus during WBRT [30, 31]. Results show that P-VMAT better spares the hippocampus compared with HT. The improved modulation ability of P-VMAT to treat volume of high dose gradient range is an advantage. Homogeneity is a common index for evaluating plan quality. With P-VMAT, the hot spot volume V_44_ in PTV-brain was only 23.3 ± 9.4%, which was significantly improved compared with HT (38.2 ± 12.9%, *p* < 0.05) and DA-VMAT (54.7 ± 8.2%, *p* < 0.05). This improvement supports the use of conventional linac for treating large-scale brain metastases since the plan quality of DA-VMAT was unacceptable for clinical practice. Figures 5 and 6 show that the V_44_ in PTV-brain varies with the number and volume of GTV. Therefore, V_44_ in PTV-brain tends to increase with an increase in the number and volume of GTV. However, it depends on the distribution, location, and relation to OARs, etc. of GTV in PTV-brain, which impacts the complexity for optimization.

Gamma knife are dedicated for radiosurgery of brain metastases, and it can not be used for whole brain radiotherapy [32]. CyberKnife with linac mounting on robotic arm are also commonly utilized for radiosurgery of metastases, but due to small aperture of collimator, it is also not suitable for the WBRT + SIB [33].

Seven arcs were used for P-VMAT with a 2.5 cm field width in this study to compare with HT as similar filed width 2.51 cm was used for HT. Actually, different numbers of arcs can be utilized for P-VMAT. About 3–7 arcs compromise the dose constraints and treatment efficiency according to our experience, however, the plan quality improves with more arcs and narrower field width for each arc. If the number of arcs are more than 7, it is usually hard for the treatment planning system Pinnacle to find a solution. On the other hand, if the number of arcs are smaller than 3, the plan quality can not be improved comparing to DA-VMAT. To ensure the coverage of prescribed dose at the joint, there must be overlapping between adjacent arcs. Actually, there are indeed some requirements for linacs, the plan quality is better with movable jaws comparing to fixed jaws and as the jaw positions of some linacs were limited to maximum 2 cm cross the central axis in Y direction, so the number of arcs were limited to max. 4 for these machines. Nevertheless, 3 or 4 arcs are sufficient for most clinical cases according to our experience.

The treatment accuracy also needs to be considered for P-VMAT. For intracranial tumor, the setup error is relatively small (within 2–3 mm) [34]. Moreover, the MLC and jaws position accuracy are also very crucial to the treatment reproducibility, which are required to be less than 1 mm. Collimator angle of 90° was tried for P-VMAT, but the dose verification gamma passing rate was relatively low measured by ArcCHECK phantom (3%/3 mm criterion < 90%). This may because that the setup error of MLC positions overlapped between adjacent subareas when the collimator angle is 90°. With collimator angle of 45°, P-VMAT plans can fulfill the requirement of gamma passing rate (3%/3 mm criterion > 95%) for clinical use. Therefore, collimator angle of 45° was utilized for planning in this study. Therefore, reasonable quality assurance procedures are very important for P-VMAT.

## Conclusions

For WBRT with SIB, P-VMAT is suitable for treating a large number of brain metastases with conventional linac and is an alternative for HT. Compared with HT, P-VMAT provides comparable coverage of whole-brain target and GTV with better conformity, lower V_44_, and better dose sparing of the hippocampus, optical nerve, optical chiasm, and spinal cord. DA-VMAT was not suitable for clinical use. The ArcCHECK phantom measurements shows the feasibility of P-VMAT for clinical treatment.

## Data Availability

All data in this study is available if it is required.

## Abbreviations

P-VMAT: Piecewise volumetric modulated arc therapy
WBRT: Whole-brain radiation therapy
SIB: Simultaneous integrated boost
HT: Helical tomotherapy
DA-VMAT: Dual arc volumetric modulated arc therapy
V_44_: The proportion of volume receiving 44 Gy
SRS: Stereotactic radiosurgery
SRT: Stereotactic fractionated radiotherapy
IMRT: Intensity-modulated radiotherapy
OAR: Organs at risk
CT: Computed tomography
MRI: Magnetic resonance imaging
GTV: Gross target volume
CTV: Clinical target volume
PTV: Planning target volume
MLC: Multi-leaf collimator
CI: Conformity indices
TVPV: The absolute volume in PTV covered by the prescription dose
TV: The absolute volume of PTV
PV: The absolute volume covered by the prescription dose
HI: Homogeneity index
D_5%_: Dose covering 5% of the target volume
D_95%_: Dose covering 95% of the target volume
D_max_: Maximum dose
D_mean_: Mean dose
MU: Monitor unit
